# Linking mercury, carbon, and nitrogen stable isotopes in Tibetan biota: Implications for using mercury stable isotopes as source tracers

**DOI:** 10.1038/srep25394

**Published:** 2016-05-06

**Authors:** Xiaoyu Xu, Qianggong Zhang, Wen-Xiong Wang

**Affiliations:** 1 Division of Life Science, The Hong Kong University of Science and Technology, Clear Water Bay, Kowloon, Hong Kong; 2 Key Laboratory of Tibetan Environment Changes and Land Surface Processes, Institute of Tibetan Plateau Research, Chinese Academy of Sciences, Beijing 100101, P. R. China; 3 State Key Laboratory of Environmental Geochemistry, Institute of Geochemistry, Chinese Academy of Sciences, Guiyang 550002, China

## Abstract

Tibetan Plateau is located at a mountain region isolated from direct anthropogenic sources. Mercury concentrations and stable isotopes of carbon, nitrogen, and mercury were analyzed in sediment and biota for Nam Co and Yamdrok Lake. Biotic mercury concentrations and high food web magnification factors suggested that Tibetan Plateau is no longer a pristine site. The primary source of methylmercury was microbial production in local sediment despite the lack of direct methylmercury input. Strong ultraviolet intensity led to extensive photochemical reactions and up to 65% of methylmercury in water was photo-demethylated before entering the food webs. Biota displayed very high Δ^199^Hg signatures, with some highest value (8.6%) ever in living organisms. The δ^202^Hg and Δ^199^Hg in sediment and biotic samples increased with trophic positions (δ^15^N) and %methylmercury. Fish total length closely correlated to δ^13^C and Δ^199^Hg values due to dissimilar carbon sources and methylmercury pools in different living waters. This is the first mercury isotope study on high altitude lake ecosystems that demonstrated specific isotope fractionations of mercury under extreme environmental conditions.

Mercury (Hg) is a major environmental concern and owns high capacity of atmospheric transport^1^. Its organic form as methylmercury (MHg) is a bioaccumulative toxicant that is readily assimilated by organisms and magnified through food webs^2^,^3^,^4^. Mercury stable isotopes have been increasingly applied as source tracers in recent years^5^,^6^,^7^,^8^,^9^,^10^. Bergquist and Blum^5^ discussed the importance of photochemical reduction in mass dependent (MDF) and independent fractionation (MIF) of aqueous Hg, and suggested to use Hg isotopes to study its biogeochemical pathways. Kwon *et al.*^11^,^12^ and Xu & Wang^13^ then studied the isotopic fractionations of different Hg species during feeding experiments, and found no evidence of Hg MIF in either metabolic processes or during trophic transfer, thereby identifying the possibility of tracking sources with Hg isotopes in fish and other biological species. Mercury isotopic compositions in diverse samples such as sediment, soil, snow, and biota from marine, estuarine, freshwater, Arctic, and terrestrial ecosystems were analyzed to identify their multiple external sources, explore MHg exposure pathways, and determine the degree of photoreduction before MHg is incorporated into the trophic webs^6^,^7^,^8^,^9^,^10^,^11^,^12^,^13^,^14^,^15^,^16^,^17^,^18^,^19^.

Tibetan Plateau (TP) is the largest and highest plateau in the world with an average elevation over 4000 m asl and a large aggregate of glaciers^20^. It is home to the Himalayas and Mount Everest and named as the ‘Third Pole’ and ‘Roof of the World’^20^. The major external sources of contaminants essentially come from atmospheric deposition given its sparse population and isolation from direct anthropogenic influences^21^,^22^,^23^. Lakes in remote mountain regions are usually worthy of study because they are sensitive indicators of surrounding pollutions, especially for highly dynamic chemicals like Hg^1^,^21^,^22^. In addition, high-latitude lakes owned distinguishable ecological features due to their large size and depth, unique location, and oligosaline characters such as freezing temperature, low pH, high ultraviolet intensity, low concentrations of organic matters, low abundance of bacteria, low nutrient status, and simple trophic webs^24^,^25^,^26^. In this study, we attempted to examine if Hg isotope fractionations are influenced or how much they are influenced by these specific environmental factors on TP, what are the bioaccumulation patterns of MHg, and what are the sources of biotic MHg especially when there was not directly transported from external sources.

Previous studies on Hg in this region mostly focused on wet deposition, precipitation, and atmospheric transport rather than trophic transfer and biomagnification^21^,^22^. Only two studies explored the Hg concentrations in fish, but none of them applied Hg stable isotopes^23^,^26^. This is the first Hg isotope study in high altitude lake ecosystem, and we investigated Hg bioaccumulations, MHg sources and exposure pathways in two representative lakes on the TP: the Nam Co Lake and Yamdrok Lake (Fig. 1). The geological setting and limnology of the Nam Co Lake have been studied due to its large size and important role in hydrogeology on the TP, but Yamdrok Lake was not studied before^25^,^27^,^28^. Sediment and biotic samples from the bottom to the top trophic positions were collected and processed for analysis of Hg concentrations and nitrogen, carbon, and Hg stable isotopes. We established a MHg magnification model and calculated its food web magnification factor (FWMF). We also plotted Hg isotope ratios against %MHg (the percentage of total Hg present as MHg) in order to identify its sources and exposure pathways. Meanwhile, intraspecies variations of Hg isotopes in fish were plotted to age (total length), food sources (δ^13^C), and odd-mass Hg isotopes (^199^Hg), giving further information on Hg biogeochemistry in the lake ecosystems on the TP and implications on the usefulness of stable Hg isotopes.

## Results

### Hg accumulation through trophic web

Mercury concentrations (THg and MHg) and %MHg increased with trophic levels (δ^15^N) in the order of sediment, plant, mosquito larvae, amphipod, fish, and Ruby shelduck egg (Fig. 2). The major chemical form of Hg was inorganic species (IHg) in the sediment and organic species (MHg) in the fish (Supplementary Table S1). Mean total Hg (THg) concentrations in *G. namensis* (143 ng/g wet weight, SD = 115, n = 32) and *G. waddelli* (108 ng/g wet weight, SD = 139, n = 26) were much lower than the human health screening value (300 ng/g wet weight, *p* < 0.001)^29^. We sampled a wide range of fish age and explored large statistical variations in their Hg concentrations and isotope ratios (Figs 2, 3, 4). MHg concentrations were positively correlated to total length (*G. namensis*: r^2^ = 0.56, *p* < 0.001; *G. waddelli*: r^2^ = 0.62, *p* < 0.001) and body weight (*G. namensis*: r^2^ = 0.68, *p* < 0.0001; *G. waddelli*: r^2^ = 0.70, *p* < 0.001). We estimated *G. namensis*’s age based on previous somatic data, which was 5 to 21 years old^30^. *G. waddelli*’s age was not reported due to the absence of relevant information.

The MHg biomagnification models were: Log_10_[MHg] = 1.20[TL]–0.98 (r^2^ = 0.95, *p* = 0.001) with food chain length of 2.21 for the Nam Co Lake, and Log_10_[MHg] = 1.34[TL]–1.62 (r^2^ = 0.94, *p* < 0.001) with food chain length of 2.16 for the Yamdrok Lake. Comparable FWMFs were estimated, which were 16.0 for the Nam Co and 21.7 for the Yamdrok Lake. MHg concentrations would increase approximately 16 and 22 fold per trophic level in the Nam Co and Yamdrok Lake, respectively. Eggs of Ruddy shelduck were excluded from this model because they cannot represent the average Hg level of an adult individual. However, there may be high risks of Hg exposure through fish consumption for Ruddy shelduck and other birds with similar diets, because their body burdens of Hg were probably high due to the elevated MHg concentrations (532 ng/g) in analyzed eggs (Supplementary Table S1).

### Hg isotopic compositions in the lake

The sediment presented low and negative δ^202^Hg values but high and positive Δ^199^Hg values, and samples from shallow waters (1 m in the Yamdrok Lake) showed higher MIF signatures compared to that from deep waters (20 m in the Nam Co Lake, Supplementary Table S1). We observed extremely high MIF signatures of Hg in fish muscle: the highest Δ^199^Hg value was 8.6 for *G. namensis* and 8.0 for *G. waddelli* (Supplementary Table S1). Significant positive relationships were explored between Hg isotope ratios and Hg contents: δ^202^Hg and Δ^199^Hg values increased with increasing MHg concentrations and %MHg in the order of plant, snail, amphipod, fish juvenile, and adults for both lakes (Fig. 3). Sediment isotopic compositions also fell on the positive trends in Fig. 3. Because biotic MHg concentrations increased with trophic levels (Fig. 2), their Hg isotope ratios were thus positively correlated to δ^15^N values. We plotted Δ^199^Hg versus Δ^201^Hg values for all biotic samples and obtained the slope of Δ^199^Hg/Δ^201^Hg, which was 1.26 ± 0.02 (1SE, r^2^ = 0.99, *p* < 0.001) for the Nam Co Lake and 1.23 ± 0.01 (1SE, r^2^ = 1.00, *p* < 0.001) for the Yamdrok Lake. Assuming experimental conditions and calculations in previous studies to apply^5^,^16^, we estimate that approximately 65% of MHg in the Nam Co Lake and 66% of MHg in Yamdrok Lake went through photochemical degradations before incorporation into food webs.

### Age-dependent isotopic compositions of Hg and C in the fish

Total length was used as surrogate of fish age due to the absence of somatic data on sampled species^31^. Mercury MIF signatures in adult fish presented large intraspecies variations and negative correlations to the age: Δ^199^Hg values in the muscle decreased with increasing total length in *G. namensis* (r^2^ = 0.71, *p* < 0.001) and *G. waddelli* (r^2^ = 0.78, *p* < 0.001, Fig. 4). Carbon isotopic compositions were also age-dependent: δ^13^C values increased with increasing total length in *G. namensis* (r^2^ = 0.67, *p* < 0.001) and *G. waddelli* (r^2^ = 0.72, *p* < 0.001, Fig. 4).

Because the carbon and odd-mass Hg isotopes were both age-dependent, we plotted δ^13^C versus Δ^199^Hg ratios and obtained obvious negative correlations as well. The Δ^199^Hg values decreased with increasing δ^13^C in both *G. namensis* (r^2^ = 0.72, *p* < 0.001) and *G. waddelli* (r^2^ = 0.82, *p* < 0.001, Fig. 4). However, we did not find any relationship between stable nitrogen (δ^15^N) and Hg (δ^202^Hg and Δ^199^Hg) isotopes in these fish.

## Discussion

This is the first Hg isotope study on high altitude lake ecosystems. Tibetan Plateau (TP) locates in an isolated high landform with minor influence from anthropogenic inputs, and the external sources primarily come from geological activities and long-range atmospheric transport^6^,^21^,^22^,^23^,^32^. Derived Hg concentrations in samples like snow, sediment, and biota from previous studies suggested that TP is likely excluded as a pristine site in terms of Hg contamination^21^,^22^,^23^,^26^,^33^. The moderately high levels of Hg in this study also indicated the same situation, though the sediment concentrations were lower than most non-TP samples and the fish concentrations did not exceed the human health screening value yet^29^,^34^,^35^,^36^,^37^. With an increasing input of external Hg^33^ and the biomagnification effect, however, Hg pollution in this region is expected to become increasingly serious in the future. For instance, a marked increase of Hg input since 1990s was observed due to the economic development in Asia, and the wet deposition flux from pollution sources of atmospheric Hg after 2000 was estimated to be 5.1 to 7.9 μg/m^2^ per year, which was similar to USA and Europe values^33^,^38^.

Lakes on the TP are considered extreme environments and unique ecosystems with distinctive characteristics. The high altitude and low DOC concentrations (4 mg/L in Nam Co Lake) result in strong ultraviolet intensity and high light penetration depth in the water^20^,^25^. Strong photochemical reactions accordingly occurred^16^,^24^,^25^,^39^,^40^: the percentage of MHg in the water that went through photochemical degradations before entering trophic webs was up to 65% in sampled lakes, which were much higher than 4–40% in Florida lakes and 5–12% in the Northeastern coast of the U.S.^15^,^16^. The plotted slopes of Δ^199^Hg/Δ^201^Hg (Nam Co: 1.26; Yamdrok: 1.23) suggested photochemical demethylation of MHg was the major process that elevated Δ^199^Hg values in the biota^5^,^7^,^14^,^16^,^17^. All sediment and biotic samples in this study presented positive MIF signatures of Hg, especially the fish muscle whose Δ^199^Hg values were highest ever compared to related studies (Supplementary Table S1)^8^,^15^,^16^,^17^,^18^. Meanwhile, photo-demethylation can increase the δ^202^Hg ratios in the substrate of MHg, leading to great positive offsets of MDF signatures in biotic MHg. Except the influences on Hg isotopic fractionations, the extensive photochemical degradations of MHg also resulted in relatively low %MHg in the water of Tibetan lakes^41^.

We observed very low δ^202^Hg values in the lake sediment (Nam Co: −2.10%; Yamdrok: −2.04%, Supplementary Table S1). Given the small percentage of MHg (<2%, Supplementary Table S1), it is IHg that presented such low δ^202^Hg signatures. Firstly, TP is a remote area with Hg generally coming from surrounding industrial activities, whose δ^202^Hg ratios were between −1.0 and 0% and higher than our samples^42^. We believe that the processes of long-distance transport and atmospheric deposition lowered the MDF signatures of input Hg before it was deposited to the lakes^21^,^22^. For example, the rain samples from Lhasa City on the TP also presented a mean δ^202^Hg value lower than 0%^43^. Secondly, the sediment δ^202^Hg values were also influenced by the net effect of methylation versus demethylation of Hg mediated by microbes in the sediment^44^,^45^. It has been reported that microbial activities could cause MDF of Hg and shift the δ^202^Hg signatures in the substrates and reactants^44^,^45^. But these processes could unlikely influence the total Hg isotope composition of sediments due to their low %MHg (<2%, Supplementary Table S1). Thirdly, extensive photochemical demethylation degraded about 65% of the MHg in the water as discussed above, driving the δ^202^Hg ratios in the product of IHg or Hg(0) to a very negative value prior to deposition^5^. This may also contribute to the low MDF signatures in our sediment samples. Additionally, watershed transport of soil particles with low δ^202^Hg values may be another reason of the specific MDF signatures in sediment IHg^6^.

Methylmercury in Tibetan lake ecosystems biomagnified with a very high efficiency. The estimated FWMFs (Nam Co: 16.0; Yamdrok: 21.7) were much higher than 5.4 in Sul River of Paraiba^46^, 4.9 and 9.5 in South River of Virginia^2^, and 10 in lower Chesapeake Bay^4^. This was caused by large difference of MHg concentrations between the bottom (plant) and top (fish) species within a relatively short food chain (<3), especially the long life span of Tibetan fish that increased MHg concentrations at the top species and thus elevated the FWMFs (Fig. 2 and Supplementary Table S1)^47^. The efficient food use and high assimilation of MHg by zooplankton also enhanced the accumulation of MHg^26^. Meanwhile, Hg isotopic compositions (Δ^199^Hg and δ^202^Hg) also increased with MHg concentrations and %MHg in the sediment and biotic samples (Fig. 3). Since results from previous studies did not support Hg MIF during trophic transfer and *in vivo* processes^11^,^12^,^13^,^44^,^45^,^48^,^49^,^50^,^51^, the observed Δ^199^Hg enrichment with trophic level was attributed to the efficient biomagnification of MHg, the varying percentage in MHg among trophic levels, and the great differences in isotopic signatures between IHg and MHg^2^,^3^,^4^,^9^,^10^,^15^,^16^,^17^,^18^. Biotic δ^202^Hg enriched approximately 3% from the sediment to the fish in both lakes, which were much higher than <1.0% in the estuarine ecosystems^15^ and <1.5% in the Florida lakes^16^. Except the reasons mentioned to explain Δ^199^Hg enrichment, this δ^202^Hg increase was also likely caused by metabolic activities because organisms tended to eliminate lighter isotopes and retain heavier isotopes^5^.

*In situ* sedimentary production of MHg was commonly suggested as the major source of bioaccumulated MHg^52^,^53^. The study of Arctic Alaska demonstrated the biogeochemical combination of IHg and sulfate reducing bacteria in near-shore deposits resulted in considerable production and mobilization of MHg to the overlying waters^54^. Here we derived a much higher δ^202^Hg values in the MHg before its photo-demethylation compared to the sediment, which was attributed to the net effect of *in situ* microbial methylation versus demethylation by previous studies^8^,^15^,^18^. Meanwhile, the δ^202^Hg and Δ^199^Hg values of sediment just fell on the regression lines of %MHg versus Hg isotopic compositions (Δ^199^Hg and δ^202^Hg) for the biota (Fig. 3), suggesting close connections between sediment and biotic MHg. *In situ* production of MHg in the water was proposed as another important source of biotic MHg recently^54^,^55^. In field and experimental studies, methylation of IHg in marine waters was mediated by abiotic and microbial mechanisms^56^,^57^,^58^,^59^,^60^. Lehnherr^56^ explored that the remineralization of particulate organic carbon drove the net methylation of IHg in polar marine waters, but other factors such as MHg demethylation and IHg availability may also affect this reaction. However, we cannot prove it due to the lack of Hg data on waters at different depths. Considering the low concentrations of organic matter in Tibetan lakes that decreased reaction potentials and the strong photochemical reductions that degraded most MHg (65.2–66.0%) in the water, we hypothesized that water methylated MHg was not the major source of biotic MHg. Moreover, watershed transport of soil particles with low δ^202^Hg values may be another source^6^, but Hg isotopic signatures in the biota and sediment did not indicate an obvious external input in Fig. 3. Consequently, microbial methylations in the sediment of Tibetan waters provided the major source of biotic MHg, while water methylation and watershed transport are minor sources.

We observed negative correlations of fish Δ^199^Hg versus age (Fig. 4), and attributed it to different living environments instead of metabolic MIF during growth, because *in vivo* MIF was not supported theoretically or experimentally until now^11^,^12^,^13^,^44^,^50^,^51^. In this study, the adult *G. namensis* and *G. waddelli* mostly occupy the main lakes except migrating for spawning from May to July every year or every two years^61^, and the juveniles usually stay in tributaries before 5 years of age where the water depth is lower (Nam Co Lakes: 2 to 93 m^28^; tributaries: 1–2 m) and the size of its MHg pool is much smaller. Because light penetration depths were similar between tributaries and lakes based on their close DOC concentrations^62^, the intraspecies variations of Hg isotopes were probably caused by different sizes of MHg pool. Rayleigh fractionation (MIF) drives river Δ^199^Hg to extreme values, but the lake Δ^199^Hg will likely not evolve to extreme values due to its relatively large MHg pool. We also observed positive correlations between fish δ^13^C and age (Fig. 4), indicating that juveniles and adults were related to low and high δ^13^C sources, respectively. Li^63^ explored the origin of carbon in the surface sediments of several TP lakes and demonstrated that organic carbon came from two sources, the autochthonous carbon from lacustrine algae, phytoplankton, and submerged plants, and the allochthonous carbon from terrestrial C3 plants with very low δ^13^C values (−33 to −24 %)^64^. Tributaries were influenced by the allochthonous carbon according to their geomorphologic features and presented lower δ^13^C signatures, whereas the main lakes were influenced by the autochthonous carbon and presented higher δ^13^C signatures. Another possible explanation was that adult fish had a greater dependence on benthic food items compared to juvenile fish. Related studies found that organisms with less negative δ^13^C values preferred to feed benthically instead of pelagically^3^,^65^. This was consistent with the feeding habits of our sampled species as the juveniles mainly feed on algae but adults feed on shrimp, snail, clam, and benthic fish like *Noemacheilus incertus*^30^. Both carbon source and feeding preference therefore contributed to the observed relationships between δ^13^C and age in the fish (Fig. 4).

The mass dependent and independent fractionations of Hg stable isotopes have been frequently applied as two-dimensional source tracers to reveal its biogeochemical pathways^6^,^7^,^9^,^10^,^15^,^16^,^17^,^18^. The MDF of Hg occurs in many situations, such as emission from anthropogenic sources^34^, physical settling in the water, microbial activities^44^,^48^,^49^, and abiotic reactions^5^,^66^,^67^, but MIF was observed only in the photoreduction of MHg or IHg since now^5^,^7^,^8^,^11^,^12^,^13^. Fish, due to its high Hg concentrations and the corresponding ecological risks, are used as representative organisms during laboratory and field experiments^11^,^12^,^13^,^15^,^16^,^17^,^18^. Here for the first time we suggested putting age into account in the application of Hg stable isotopes in fish, especially for the migrating species and under strong ultraviolet conditions. Different environments own dissimilar Hg pools and light penetration depths that strongly influence the degree of photochemical reactions in the water. Such influence will be substantially enhanced in high-latitude lakes where the ultraviolet is much stronger than other ecosystems. In this study, we explored a wide range of Hg concentrations and isotopic compositions for *G. namensis* in the Nam Co Lake and *G. waddelli* in the Yamdrok Lake, especially the elevated Δ^199^Hg values in fish muscle (Figs 2, 3, 4). The anomalous MIF signatures, such as higher Δ^199^Hg values in organisms at low trophic level compared to top predators like fish (Fig. 3), may be caused by different environmental factors rather than input of some unknown sources. Thus, selecting appropriate organisms is one significant thing in using Hg stable isotopes as source tracers. When a migrating species is selected, fish age, living waters, and the corresponding environmental factors all need to be considered.

Carbon stable isotope ratios (δ^13^C) have been widely used to infer food sources, and many studies explored the relationships between Hg and δ^13^C values^68^,^69^. It is well known that biota accumulated Hg through diet, which is consequently related to food or carbon sources. In this study, the δ^13^C values decreased with increasing MIF signatures of Hg in adult fish (Fig. 4), meaning that juveniles living in tributaries presented low δ^13^C and high Δ^199^Hg values but adults living in major lakes presented high δ^13^C and low Δ^199^Hg values. We did not find any relationship between nitrogen (δ^15^N) and Hg isotopic compositions (Δ^199^Hg and δ^202^Hg), suggesting that food source rather than trophic uptake dominated Hg isotope ratios in Tibetan fish. In general, Hg isotopic signatures in aquatic biota on the TP were primarily controlled by ultraviolet intensity, food (carbon) sources, and the size of MHg pool. It has been argued that the MDF and MIF of Hg isotope ratios are inadequate to uniquely identify each source of Hg when many different external inputs exist at the same time^70^. Here, we explored good correlations between carbon and Hg isotopes in the fish (Fig. 4), which revealed potential links among different isotopes on the same organism and suggested the possibilities of using δ^13^C signature as a supplement in such studies^68^. The combined results of different isotopes will provide more accurate indications on Hg sources and become a better tool in understanding the life histories of fish and other organisms.

## Methods

### Field sampling

**Figure 1:**
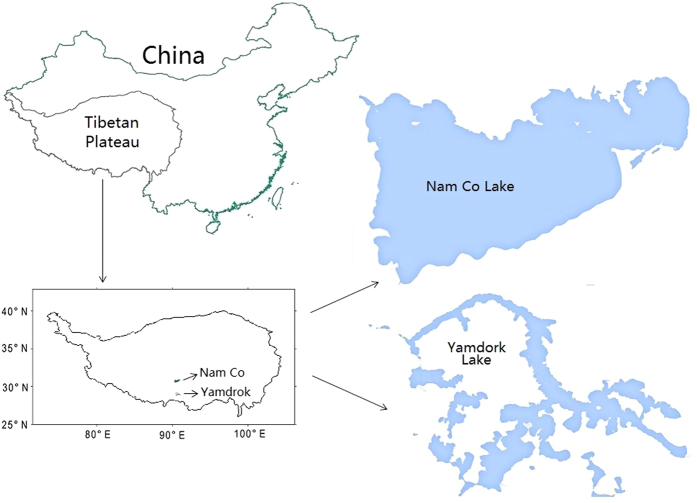
Map of sampling sites at the Nam Co and Yamdrok Lake on the southern Tibetan Plateau of China. All four items were generated with Adobe Illustrator CS4.

**Figure 2:**
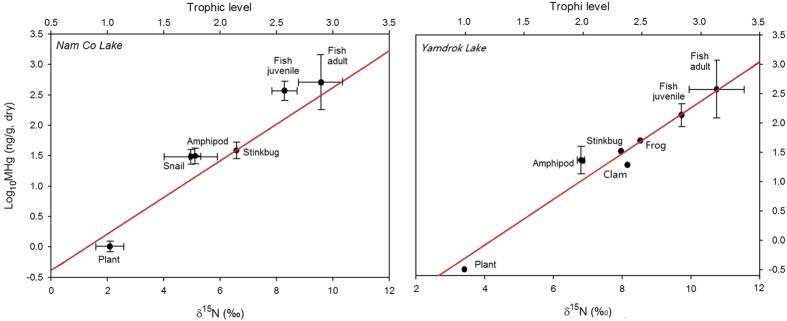
Influence of trophic position and δ^15^N values on methylmercury accumulation among sampled species in the Nam Co and Yamdrok Lake. Filled circle and error bar on individual data point reflect means and 1 SD of field sampling replicates from the same lake.

**Figure 3:**
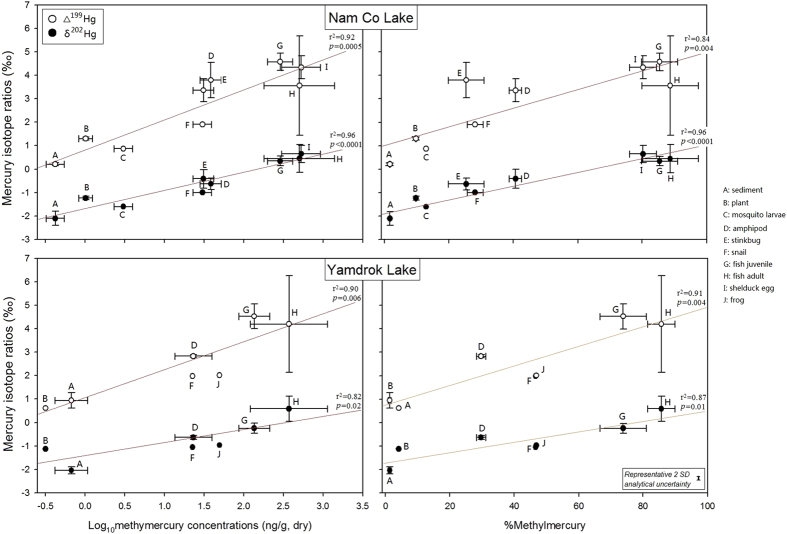
Relationships of mercury contents (methylmercury concentrations and %mehylmercury) and isotopic compositions (δ^202^Hg and Δ^199^Hg) for sediment and biotic samples in the Nam Co and Yamdrok Lake. Circles and error bars on individual data point reflect means and 1 SD of field sampling replicates from the same lake. Representative 2 SD of analytical uncertainty during mercury isotope analysis is shown based on measurement of procedural standards. Capital English letters represent different samples: A, sediment; B, plant; C, mosquito larvae; D, amphipod; E, stinkbug; F, snail; G, fish juvenile; H, fish adult; I, shelduck egg; J, frog.

**Figure 4:**
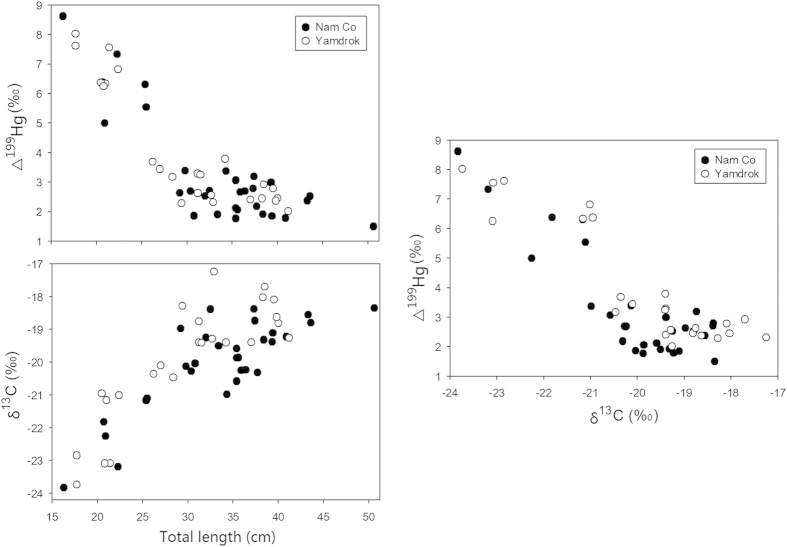
The relationship among Δ^199^Hg, δ^13^C, and total body length in fish adult. Filled circle stands for fish from Nam Co Lake, and open circle stands for fish from Yamdrok Lake.

Two lakes on the southern Tibetan Plateau (TP) were sampled during the early summer of 2014 (Fig. 1). Nam Co Lake, located at the Nyainqentanglha mountain range, is a large saltwater lake covering an area of 2015 km^2^ at the average elevation of 4718 m^29^. Yamdrok Lake, located at the northern range of the Himalayas, is a fan-shaped saltwater lake covering an area of 650 km^2^ at the average elevation of 4441 m^71^. Surface sediment (upper 5–10 cm), plant (*Potamogeton pectinatus*), mosquito (*Chironomidae*) larvae, amphipod (*Gammaridea*), backswimmers (*Notonectidae*), snail (*Radix sp.*), juvenile and adult fish (*Gymnocypris namensis* from Nam Co Lake, and *Gymnocypris waddelli* from Yamdrok Lake) were collected at both lakes. Meanwhile, the ruddy shelduck (*Tadorna ferruginea*) eggs from the Nam Co Lake, and clams (*Sphaerium sp.*), dragonflies, and frogs from the Yamdrok Lake were sampled. We collected all biotic samples but fish at replicated sites that were randomly chosen from each lake. The fish samples were caught at tributaries instead of the main lake because they were migrating for spawning during the sampling occasion. The total body length of fish juveniles fluctuated around 5 cm, but the ranges were much wider for adult fish (*G. namensis*: 16.3 to 50.6 cm; *G. waddelli*: 17.7 to 41.2 cm). Only 3 sediment samples from the Nam Co Lake (at water depth of 20 m) and 2 from the Yamdrok Lake (at water depth of 1 m) were collected due to the difficulties and limitations of field work at high-latitude mountain regions.

### Ethics statement

All the methods were carried out in accordance with the approved guidelines. In addition, all the experimental protocols were approved by the Hong Kong University of Science and Technology safety committee.

### Sample preparation

Biotic samples were cleaned with Milli-Q water, identified, and stored at −20 °C until further processing. Sediment samples were kept refrigerated in the dark all the time. The axial muscle of each adult fish was dissected after determining the body weight, length, and sex. Fish muscle was used due to its high percentage of methylmercury (MHg). We focused on Hg trophic transfer instead of Hg redistribution during metabolic activities, thus only muscle was analyzed in this study. Eggshells of Ruddy shelducks’ egg were opened and the yolk fractions were separated from album. Subsequently, all samples were freeze dried at −80 °C (Freeze-Dryer, ilShinBioBase Co. Ltd., South Korea) for a week, and the weights were measured before and after the freeze drying to calculate the wet to dry weight quotients. Individuals of similar size within a sampling site were pooled to produce replicates or triplicates for plants, amphipods, mosquito larvae, backswimmers, clams, and fish juveniles because of their small body sizes and low levels of Hg. The mentioned concentrations in this study were based on dry weight unless wet weight was noted.

### Mercury analysis

Total mercury concentrations (ng/g dry weight) were analyzed by cold vapor atomic fluorescence spectrometry (Quick Trace M-8000, CETAC Technologies, USA). Dried biotic samples were digested with ultrapure acid (H_2_SO_4_:HNO_3_ = 1:4, v/v, Sigma, USA) on block heater (BT5D, Grant Instruments, UK) at 95 °C for 3 to 4 hours^72^. Mercury of the sediment samples were extracted with mixed acid (H_2_SO_4_:HNO_3_ = 4:5, v/v) for 24 hour^18^. Aliquots of the digested samples were appropriately diluted, oxidized to Hg(II) with a hydrochloride/bromate/bromide mixture, reduced with hydroxylamine hydrochloride to destroy the free halogens, and converted to Hg(0) with stannous chloride^73^. The Hg(0) vapor was then separated from solution by purging with nitrogen, collected by gold traps, and thermally desorbed from the analytical trap into a fluorescence detector. The MHg concentration was analyzed by an automated MHg analytical system (MERX, Brooks Rand, USA). Samples were digested with alkaline (25% KOH in 10% methanol) at 75 ^o^C for 3 hours in closed vials, and appropriate aliquots were buffered with sodium acetate to pH 4.9 and ethylated by sodium tetraethylborate^74^,^75^. The quantification of MHg was automatically performed by the gas chromatographic separation and pyrolysis, following the atomic fluorescence detection. Standard curves were established using a liquid Hg standard (PerkinElmer, USA) for THg analysis and CH_3_HgCl (Brooks Rand, Washington, USA) for MHg analysis within each analytical session. Precision and accuracy for the analytical system were quantified with blanks, 10% replicated samples, and 10% standard reference material DORM-4 (fish protein, National Research Council of Canada) and IAEA-452 (scallop, international Atomic Energy Agency). Precision expressed as relative percent difference for duplicate samples averaged 3.0 (SD = 2.4%, n = 22) for THg analysis and 4.0 (SD = 3.5%, n = 21) for MHg analysis. The mean percent recoveries of DORM-4 standards were 99.6 (SD = 6.7%, n = 45) for THg analysis and 97.2 (SD = 8.2%, n = 42) for MHg analysis, and of IAEA-452 standards were 97.3 (SD = 4.2%, n = 22) for THg analysis and 98.1 (SD = 5.6%, n = 20) for MHg analysis. Inorganic Hg (IHg) was not analyzed, because it is not the major bioaccumulated species and its percentage in total Hg was very low in fish.

### Nitrogen and carbon isotopes

The δ^15^N and δ^13^C in sediment, fish muscle and other biotic samples were determined at the stable isotope facility of the University of California (Davis, CA). An appropriate portion of freeze-dried and ground sample were weighed and compressed into tin capsules. The δ^15^N and δ^13^C were analyzed with a PDZ Europa ANCA-GSL elemental analyzer interfaced with a PDZ Europa 20–20 isotope ratio mass spectrometer. Results were expressed as ratios per mil (‰) normalized to isotopic composition of Pee Dee Belemnite Limestone (δ^13^C) or atmospheric N_2_ (δ^15^N) standards. Analytical accuracy and precision were assessed using recoveries and associated standard deviations for replicate analyses of four standard reference materials: G-13 (bovine liver), G-17 (USGS-41 glutamic acid), G-18 (nylon 5), and G-20 (glutamic acid). The δ^13^C mean percent recovery of G-13, G-17, G-18, and G-20 were 99.7% (SD = 0.2%, n = 2), 100.0% (SD = 0.1%, n = 4), 100.0% (SD = 0.08%, n = 21), and 99.9% (SD = 0.08%, n = 5), respectively. The δ^15^N mean percent recovery of G-9, G-11, G-12, G-13, and G-17 were 100.0% (SD = 0.07%, n = 2), 100.0% (SD = 0.1%, n = 4), 100.0% (SD = 0.08%, n = 24), and 100.4% (SD = 0.1%, n = 6), respectively.

### Methylmercury magnification model

Conventional magnification models with MHg concentrations and estimated relative trophic levels for sampled species in both lakes were established using the Regression procedure in SigmaPlot 13.0 (Systat Software Inc., USA). The trophic level (TL) for each species were estimated from its δ^15^N values assuming the trophic level of plant (*Potamogeton pectinatus*) was 1, and that δ^15^N increased 3.4% per TL^76^. Therefore, the trophic level of a species can be calculated with Equation (1). The length of the studied food web was determined as the difference between the highest and lowest trophic level for the species considered^77^. A linear regression model was fit between TLs and log10-transformed MHg concentrations (dry weight) for sampled species using Equitation (2). The food web magnification factor (FWMF) was estimated with Equation (3), where “a” is the slope from the linear regression in Equation 2^2^,^4^.


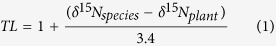



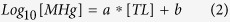



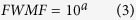


### Mercury stable isotopes

An aliquot of sample solution for THg analysis with the mass of 45 ng THg was removed and diluted to 15 mL by adding Milli-Q water; for sample solutions with the Hg mass lower than 45 ng, 30 ng or 15 ng of THg was removed and diluted to 15 mL^72^. Mercury isotopic ratios were determined by MC-ICP-MS (Nu Plasma HR, Nu Instruments, Great Britain) with a modified cold vapor generation system that introduced Hg to the detection system^78^. The internal standard thallium (Tl) was added to the Hg vapor with a desolvating nebulizer (DSN-100, Nu Instruments, Great Britain), and the Hg isotope ratios were corrected for instrumental mass discrimination by simultaneously monitoring the ^205^Tl/^203^Tl ratio of a standard solution. Instrumental mass bias was also corrected using a sample-standard bracketing with NIST 3133 (in 2% HCl), whose concentration and matrix matched with that of the samples during the analysis. The instrumental setup, analytical conditions and parameters determinations followed previous studies^72^. Mercury isotopic compositions were reported in delta notation as permil (%) deviation from NIST 3133 Hg standard with the equation (4)^5^, where xxx is mass of each Hg isotope between 199 and 202. The δ^202^Hg values were reported to indicate MDF in this study, and the MIF signatures were reported in capital delta notation (Δ^xxx^Hg) using equations (5) and (6)^5^.


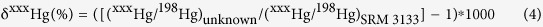



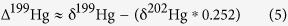



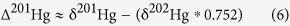


The analytical uncertainty was quantified by analysis of standard reference materials and result for each sample was expressed with 2 SD typical errors in replicates. The mean values and 2 SDs of UM-Almáden were −0.58 ± 0.07% for δ^202^Hg, −0.05 ± 0.05% for Δ^201^Hg, and −0.03 ± 0.07% for Δ^199^Hg (n = 24); the mean values and 2 SDs of DORM-2 were 0.15 ± 0.11% for δ^202^Hg, 0.89 ± 0.06% for Δ^201^Hg, and 1.01 ± 0.05% for Δ^199^Hg (n = 7), which corresponded to previous studies^5^,^79^. When the calculated 2 SD of a sample replicate was smaller than the replicate analyses of the standard reference material, the uncertainty of the reference material was used instead.

## Additional Information

**How to cite this article**: Xu, X. *et al.* Linking mercury, carbon, and nitrogen stable isotopes in Tibetan biota: Implications for using mercury stable isotopes as source tracers. *Sci. Rep.* **6**, 25394; doi: 10.1038/srep25394 (2016).

## Supplementary Material

Supplementary Information
