# Supplementary material for: Linking mercury, carbon, and nitrogen stable isotopes in Tibetan biota: Implications for using mercury stable isotopes as source tracers
Source: Sci Rep. 2016 May 6;6:25394. doi: 10.1038/srep25394 (PMC4858742; doi:10.1038/srep25394)
Supplement: Supplementary Information [file srep25394-s1.doc]

**Linking mercury, carbon, and nitrogen stable isotopes in Tibetan biota: Implications for using mercury stable isotopes as source tracers**

Xiaoyu Xu^1^, Qianggong Zhang^2^, Wen-Xiong Wang^1,3*^

*^1^Division of Life Science, The Hong Kong University of Science and Technology, Clear Water Bay, Kowloon, Hong Kong*

*^2^Key Laboratory of Tibetan Environment Changes and Land Surface Processes, Institute of Tibetan Plateau Research, Chinese Academy of Sciences, Beijing 100101, PR. China*

*^3^State Key Laboratory of Environmental Geochemistry, Institute of Geochemistry, Chinese Academy of Sciences, Guiyang 550002, China*

**Supplementary Table S1: mercury concentrations and stable isotope ratios of mercury, carbon, nitrogen for sediment and biotic samples**

| **Sample** | **Species** | **Site** | **Total Hg** | **Methyl-Hg** | **%Methyl-Hg** | **δ^202^Hg** | **Δ^199^Hg** | **Δ^201^Hg** | **δ^13^C** | **δ^15^N** | **Length** | **Weight** | **Sex** |
| --- | --- | --- | --- | --- | --- | --- | --- | --- | --- | --- | --- | --- | --- |
|  |  |  | **(ng/g, dry)** | **(ng/g, dry)** |  | **(‰)** | **(‰)** | **(‰)** | **(‰)** | **(‰)** | **(cm)** | **(g)** |  |
| Fish-1 | *Gymnocypris waddelli* | Y | 100.5 | 79.4 | 79.0 | 0.95 | 4.91 | 4.09 | / | / | 23.5 | 87.2 | M |
| Fish-2 | *Gymnocypris waddelli* | Y | 87.4 | 70.3 | 80.4 | 1.33 | 7.56 | 6.18 | -23.08 | 10.50 | 21.4 | 64.5 | M |
| Fish-3 | *Gymnocypris waddelli* | Y | 74.2 | 69.1 | 93.1 | 1.25 | 7.53 | 6.22 | / | / | 19.3 | 78.7 | M |
| Fish-4 | *Gymnocypris waddelli* | Y | 182.9 | 157.4 | 86.1 | 1.21 | 6.34 | 5.23 | -21.16 | 9.89 | 21.0 | 35.2 | F |
| Fish-5 | *Gymnocypris waddelli* | Y | 280.8 | 241.6 | 86.0 | 0.45 | 3.29 | 2.87 | -19.39 | 10.32 | 31.2 | 204.9 | M |
| Fish-9 | *Gymnocypris waddelli* | Y | 2796.0 | 2220.8 | 79.4 | -0.10 | 2.44 | 1.99 | -18.03 | 11.49 | 38.3 | 630.0 | F |
| Fish-12 | *Gymnocypris waddelli* | Y | 256.9 | 230.2 | 89.6 | 0.40 | 3.25 | 2.68 | -19.41 | 10.40 | 31.5 | 238.5 | M |
| Fish-14 | *Gymnocypris waddelli* | Y | 649.8 | 608.6 | 93.7 | 0.37 | 2.78 | 2.44 | -18.09 | 10.91 | 39.5 | 560.0 | M |
| Fish-15 | *Gymnocypris waddelli* | Y | 1972.0 | 1584.2 | 80.3 | 0.04 | 2.45 | 2.00 | -18.82 | 12.10 | 40.0 | 770.0 | F |
| Fish-16 | *Gymnocypris waddelli* | Y | 608.6 | 504.5 | 82.9 | -0.10 | 2.37 | 1.99 | -18.63 | 11.93 | 39.8 | 470.0 | M |
| Fish-17 | *Gymnocypris waddelli* | Y | 431.4 | 381.9 | 88.5 | 0.14 | 2.92 | 2.49 | -17.70 | 12.10 | 38.5 | 375.0 | M |
| Fish-18 | *Gymnocypris waddelli* | Y | 511.7 | 423.6 | 82.8 | 0.24 | 2.01 | 1.78 | -19.26 | 9.89 | 41.2 | 335.0 | M |
| Fish-19 | *Gymnocypris waddelli* | Y | 302.2 | 255.2 | 84.4 | -0.34 | 2.40 | 2.05 | -19.39 | 10.19 | 37.0 | 310.0 | M |
| Fish-20 | *Gymnocypris waddelli* | Y | 201.0 | 169.8 | 84.5 | -0.11 | 2.32 | 1.89 | -17.24 | 9.29 | 32.9 | 275.0 | F |
| Fish-21 | *Gymnocypris waddelli* | Y | 686.1 | 568.4 | 82.8 | 0.17 | 3.78 | 3.06 | -19.40 | 12.09 | 34.2 | 285.0 | M |
| Fish-22 | *Gymnocypris waddelli* | Y | 163.6 | 131.2 | 80.2 | 0.23 | 2.62 | 2.22 | -18.76 | 11.20 | 31.2 | 235.0 | M |
| Fish-23 | *Gymnocypris waddelli* | Y | 86.3 | 69.1 | 80.2 | 0.88 | 3.44 | 2.98 | -20.10 | 9.32 | 27.0 | 180.0 | F |
| Fish-24 | *Gymnocypris waddelli* | Y | 793.0 | 723.8 | 91.3 | 0.53 | 2.28 | 1.94 | -18.28 | 11.95 | 29.4 | 185.0 | M |
| Fish-25 | *Gymnocypris waddelli* | Y | 552.1 | 472.4 | 85.6 | 0.34 | 3.17 | 2.68 | -20.47 | 10.48 | 28.4 | 165.0 | M |
| Fish-26 | *Gymnocypris waddelli* | Y | 146.3 | 134.0 | 91.6 | 0.49 | 2.56 | 2.17 | -19.29 | 10.40 | 32.7 | 130.0 | M |
| Fish-27 | *Gymnocypris waddelli* | Y | 188.1 | 168.6 | 89.6 | 0.84 | 3.68 | 3.13 | -20.35 | 10.44 | 26.2 | 115.0 | F |
| Fish-28 | *Gymnocypris waddelli* | Y | 78.9 | 65.1 | 82.5 | 1.05 | 7.61 | 6.31 | -22.85 | 10.37 | 17.7 | 70.0 | M |
| Fish-29 | *Gymnocypris waddelli* | Y | 125.6 | 109.1 | 86.9 | 1.36 | 6.82 | 5.69 | -21.01 | 10.18 | 22.4 | 70.0 | M |
| Fish-30 | *Gymnocypris waddelli* | Y | 116.9 | 109.2 | 93.4 | 1.38 | 8.02 | 6.51 | -23.73 | 11.10 | 17.7 | 70.0 | M |
| Fish-31 | *Gymnocypris waddelli* | Y | 127.7 | 118.6 | 92.8 | 1.18 | 6.37 | 5.25 | -20.95 | 10.74 | 20.5 | 70.0 | M |
|  |  |  |  |  |  |  |  |  |  |  |  |  |  |
| **Sample** | **Species** | **Site** | **Total Hg** | **Methyl-Hg** | **%Methyl-Hg** | **δ^202^Hg** | **Δ^199^Hg** | **Δ^201^Hg** | **δ^13^C** | **δ^15^N** | **Length** | **Weight** | **Sex** |
|  |  |  | **(ng/g, dry)** | **(ng/g, dry)** |  | **(‰)** | **(‰)** | **(‰)** | **(‰)** | **(‰)** | **(cm)** | **(g)** |  |
| Fish-31B | *Gymnocypris waddelli* | Y | 47.5 | 38.5 | 81.0 | 1.14 | 6.25 | 5.07 | -23.09 | 10.58 | 20.8 | 70.0 | M |
| Fish-32 | *Gymnocypris namensis* | N | 1076.1 | 959.7 | 89.2 | 0.39 | 1.78 | 1.56 | -19.23 | 9.44 | 40.9 | 550.0 | F |
| Fish-33 | *Gymnocypris namensis* | N | 398.8 | 330.4 | 82.8 | 0.39 | 3.19 | 2.72 | -18.74 | 9.59 | 37.4 | 415.0 | F |
| Fish-34 | *Gymnocypris namensis* | N | 489.0 | 406.4 | 83.1 | 0.44 | 1.91 | 1.70 | -19.32 | 9.17 | 38.4 | 450.0 | F |
| Fish-35 | *Gymnocypris namensis* | N | 375.3 | 320.7 | 85.4 | 0.11 | 2.99 | 2.51 | -19.39 | 10.13 | 39.3 | 585.0 | F |
| Fish-36 | *Gymnocypris namensis* | N | 117.4 | 105.5 | 89.8 | 0.61 | 3.39 | 2.88 | -20.13 | 9.41 | 29.8 | 200.0 | F |
| Fish-37 | *Gymnocypris namensis* | N | 1243.3 | 1100.7 | 88.5 | 0.06 | 2.37 | 2.06 | -18.55 | 9.64 | 43.3 | 670.0 | F |
| Fish-38 | *Gymnocypris namensis* | N | 1073.5 | 900.2 | 83.9 | 0.17 | 1.77 | 1.54 | -19.87 | 10.60 | 35.4 | 420.0 | F |
| Fish-39 | *Gymnocypris namensis* | N | 544.7 | 482.1 | 88.5 | -0.43 | 1.90 | 1.56 | -19.51 | 9.55 | 33.4 | 340.0 | F |
| Fish-40 | *Gymnocypris namensis* | N | 934.6 | 814.3 | 87.1 | -1.23 | 1.49 | 1.25 | -18.35 | 8.91 | 50.6 | 665.0 | F |
| Fish-41 | *Gymnocypris namensis* | N | 869.5 | 816.3 | 93.9 | -0.20 | 2.52 | 2.13 | -18.80 | 10.29 | 43.6 | 585.0 | F |
| Fish-42 | *Gymnocypris namensis* | N | 1217.2 | 1108.8 | 91.1 | 0.19 | 2.18 | 1.91 | -20.32 | 9.50 | 37.7 | 460.0 | F |
| Fish-43 | *Gymnocypris namensis* | N | 1707.5 | 1591.4 | 93.2 | 0.13 | 2.11 | 1.86 | -19.59 | 9.84 | 35.4 | 375.0 | M |
| Fish-44 | *Gymnocypris namensis* | N | 280.3 | 250.5 | 89.4 | 0.62 | 2.69 | 2.29 | -20.28 | 8.76 | 30.4 | 260.0 | M |
| Fish-45 | *Gymnocypris namensis* | N | 449.9 | 411.0 | 91.3 | 0.30 | 2.06 | 1.78 | -19.86 | 10.26 | 35.6 | 305.0 | M |
| Fish-46 | *Gymnocypris namensis* | N | 1124.7 | 1051.6 | 93.5 | 0.31 | 2.69 | 2.29 | -20.24 | 9.80 | 36.4 | 485.0 | F |
| Fish-47 | *Gymnocypris namensis* | N | 283.3 | 263.1 | 92.8 | 0.49 | 2.63 | 2.24 | -18.97 | 8.99 | 29.2 | 230.0 | F |
| Fish-48 | *Gymnocypris namensis* | N | 769.2 | 655.8 | 85.3 | 0.30 | 2.53 | 2.12 | -19.25 | 9.20 | 32.0 | 260.0 | M |
| Fish-49 | *Gymnocypris namensis* | N | 1363.5 | 1029.2 | 75.5 | 0.16 | 2.67 | 2.20 | -20.25 | 10.48 | 35.9 | 340.0 | M |
| Fish-50 | *Gymnocypris namensis* | N | 736.7 | 617.5 | 83.8 | 0.16 | 1.86 | 1.56 | -20.04 | 9.28 | 30.8 | 245.0 | M |
| Fish-51 | *Gymnocypris namensis* | N | 131.3 | 113.8 | 86.6 | 0.86 | 5.54 | 4.41 | -21.11 | 9.89 | 25.5 | 135.0 | F |
| Fish-52 | *Gymnocypris namensis* | N | 58.3 | 49.3 | 84.6 | 0.81 | 5.00 | 4.06 | -22.26 | 9.01 | 20.9 | 75.0 | M |
| Fish-53 | *Gymnocypris namensis* | N | 30.9 | 26.8 | 86.7 | 0.55 | 3.06 | 2.57 | -20.58 | 7.08 | 35.4 | 115.0 | M |
| Fish-54 | *Gymnocypris namensis* | N | 85.3 | 69.4 | 81.4 | 1.57 | 8.43 | 6.82 | / | / | 18.2 | 70.0 | M |

| **Sample** | **Species** | **Site** | **Total Hg** | **Methyl-Hg** | **%Methyl-Hg** | **δ^202^Hg** | **Δ^199^Hg** | **Δ^201^Hg** | **δ^13^C** | **δ^15^N** | **Length** | **Weight** | **Sex** |
| --- | --- | --- | --- | --- | --- | --- | --- | --- | --- | --- | --- | --- | --- |
|  |  |  | **(ng/g, dry)** | **(ng/g, dry)** |  | **(‰)** | **(‰)** | **(‰)** | **(‰)** | **(‰)** | **(cm)** | **(g)** |  |
| Fish-55 | *Gymnocypris namensis* | N | 92.3 | 78.3 | 84.9 | 1.25 | 6.38 | 5.23 | -21.82 | 8.66 | 20.7 | 70.0 | M |
| Fish-56 | *Gymnocypris namensis* | N | 92.9 | 81.3 | 87.6 | 1.51 | 7.69 | 6.24 | / | / | 17.6 | 75.0 | M |
| Fish-57 | *Gymnocypris namensis* | N | 117.6 | 105.2 | 89.4 | 1.22 | 7.33 | 5.97 | -23.19 | 9.58 | 22.3 | 95.0 | M |
| Fish-58 | *Gymnocypris namensis* | N | 86.3 | 77.9 | 90.3 | 1.63 | 8.62 | 6.94 | -23.83 | 10.10 | 16.3 | 70.0 | M |
| Fish-59 | *Gymnocypris namensis* | N | 395.5 | 371.8 | 94.0 | 0.45 | 2.71 | 2.24 | -18.39 | 10.54 | 32.5 | 280.0 | F |
| Fish-60 | *Gymnocypris namensis* | N | 95.8 | 81.2 | 84.7 | 1.15 | 6.31 | 5.16 | -21.16 | 9.35 | 25.4 | 110.0 | F |
| Fish-61 | *Gymnocypris namensis* | N | 396.3 | 386.1 | 97.4 | 0.04 | 3.37 | 2.85 | -20.98 | 10.93 | 34.3 | 405.0 | F |
| Fish-62 | *Gymnocypris namensis* | N | 753.4 | 718.4 | 95.4 | 0.04 | 2.79 | 2.32 | -18.38 | 11.29 | 37.3 | 420.0 | F |
| Fish-63 | *Gymnocypris namensis* | N | 875.3 | 829.6 | 94.8 | 0.28 | 1.85 | 1.62 | -19.11 | 9.54 | 39.4 | 500.0 | F |

| **Sample** | **Species** | **Site** | **Total Hg** | **Methyl-Hg** | **%Methyl-Hg** | **δ^202^Hg** | **Δ^199^Hg** | **Δ^201^Hg** | **δ^13^C** | **δ^15^N** |
| --- | --- | --- | --- | --- | --- | --- | --- | --- | --- | --- |
|  |  |  | **(ng/g, dry)** | **(ng/g, dry)** |  | **(‰)** | **(‰)** | **(‰)** | **(‰)** | **(‰)** |
| Sediment-1 | */* | Y | 56.8 | 0.9 | 1.6 | -1.93 | 1.17 | 1.17 | -4.44 | 3.42 |
| Sediment-5 | */* | Y | 45.1 | 0.5 | 1.0 | -2.15 | 0.71 | 0.35 | -4.40 | 3.62 |
| Sediment-2 | */* | N | 23.2 | 0.4 | 1.7 | -2.34 | 0.25 | 0.28 | -5.29 | 2.47 |
| Sediment-3 | */* | N | 25.6 | 0.4 | 1.7 | -2.20 | 0.23 | 0.15 | -7.00 | 3.18 |
| Sediment-4 | */* | N | 29.7 | 0.5 | 1.5 | -1.75 | 0.14 | 0.13 | -2.73 | 1.97 |
| Plant-1 | *Potamogeton pectinatus* | N | 11.8 | 1.2 | 9.8 | -1.18 | 1.34 | 1.68 | -7.17 | 2.44 |
| Plant-2 | *Potamogeton pectinatus* | N | 9.1 | 0.9 | 9.6 | -1.29 | 1.25 | 1.00 | -7.27 | 1.74 |
| Plant-3 | *Potamogeton pectinatus* | Y | 7.7 | 0.3 | 4.1 | -1.13 | 0.61 | 0.66 | -10.48 | 3.41 |
| Mosquito (larvae)-1 | *Chironomidae* | N | 34.3 | 4.4 | 12.8 | / | / | / | / | / |
| Mosquito (larvae)-2 | *Chironomidae* | N | 13.1 | 1.7 | 12.9 | -1.60 | 0.87 | 0.82 | / | / |
| Mosquito (larvae)-3 | *Chironomidae* | Y | 11.4 | 0.6 | 5.2 | / | / | / | / | / |
| Amphipod-1 | *Gammaridea* | N | 63.7 | 29.7 | 46.6 | -0.70 | 3.71 | 3.07 | -19.77 | 5.01 |
| Amphipod-2 | *Gammaridea* | N | 89.6 | 32.8 | 36.6 | -0.12 | 3.01 | 2.46 | -15.78 | 5.23 |
| Amphipod-3 | *Gammaridea* | Y | 86.2 | 26.2 | 30.4 | -0.58 | 2.79 | 2.22 | -27.14 | 6.87 |
| Amphipod-4 | *Gammaridea* | Y | 70.4 | 20.2 | 28.7 | -0.69 | 2.87 | 2.10 | -26.58 | 6.73 |
| Snail-1 | *Radix sp.* | N | 91.4 | 27.8 | 30.4 | -1.00 | 1.90 | 1.64 | -18.91 | 4.29 |
| Snail-2 | *Radix sp.* | N | 123.6 | 32.6 | 26.4 | / | / | / | -18.39 | 5.62 |
| Snail-3 | *Radix sp.* | Y | 48.6 | 22.7 | 46.6 | -1.05 | 1.97 | 1.80 | -23.34 | 6.85 |
| Stinkbug-1 | *Notonectidae* | N | 162.0 | 40.2 | 24.8 | -0.45 | 4.33 | 3.53 | -20.04 | 6.58 |
| Stinkbug-2 | *Notonectidae* | N | 141.9 | 36.7 | 25.8 | -0.81 | 3.26 | 3.38 | / | / |
| Stinkbug-3 | *Notonectidae* | Y | 83.4 | 32.8 | 39.4 | / | / | / | -29.18 | 7.96 |
| Clam | *Sphaerium sp.* | Y | 43.3 | 19.1 | 44.0 | / | / | / | -29.88 | 8.15 |
| Dragonfly | */* | Y | 52.9 | 31.7 | 60.0 | / | / | / | -29.68 | 9.83 |
| Frog | */* | Y | 105.8 | 49.5 | 46.8 | -0.97 | 2.01 | 1.67 | -22.24 | 8.53 |
